# Precipitation Alters the Effects of Temperature on the Ecosystem Multifunctionality in Alpine Meadows

**DOI:** 10.3389/fpls.2021.824296

**Published:** 2022-02-09

**Authors:** Yunhe Ma, Lihua Tian, Guangpeng Qu, Ruicheng Li, Weiwei Wang, Jingxue Zhao

**Affiliations:** ^1^State Key Laboratory of Grassland Agro-Ecosystems, Institute of Innovation Ecology and College of Life Sciences, Lanzhou University, Lanzhou, China; ^2^State Key Laboratory of Hulless Barley and Yak Germplasm Resources and Genetic Improvement, Grassland Science Institute, Tibet Academy of Agricultural and Animal Husbandry Sciences, Lhasa, China; ^3^Institute of Qinghai-Tibetan Plateau, Southwest Minzu University, Chengdu, China; ^4^Key Laboratory for Earth Surface Processes of the Ministry of Education, Department of Ecology, College of Urban and Environmental Sciences, Peking University, Beijing, China; ^5^Key Laboratory of Plateau Mountain Animal Genetics, Breeding and Reproduction, Ministry of Education, College of Animal Science, Guizhou University, Guiyang, China

**Keywords:** alpine meadow, ecosystem multifunctionality, climate change, reciprocal transplantation, Tibetan Plateau

## Abstract

Precipitation and temperature are major controls on multiple ecosystem functions in alpine grasslands. There is scant evidence for the interactive effects of temperature and precipitation changes on the ecosystem multifunctionality (EMF) in alpine meadows. To explore the interactive effects of temperature and precipitation changes on the EMF in alpine meadows, we transplanted meadow blocks reciprocally among three altitudes (4,650, 4,950, and 5,200 m) on the central Tibetan Plateau. Compared with the home sites (control), the EMF has a trend to increase when meadow blocks were downward transplanted (experimental warming) to the high-precipitation sites but decrease as meadow blocks were downward transplanted to the low-precipitation sites. However, the experimental cooling (upward transplantation) consistently reduced the EMF regardless of the precipitation change. The increase of EMF under the experimental warming was closely related to the variation of both plant and soil functions, whereas the reduction of EMF under the cooling was highly correlated with the decrease of plant function. Our results highlight that climate warming effects on the EMF are greatly associated with precipitation changes in the semi-arid alpine ecosystems.

## Highlights

- Cooling consistently reduces EMF regardless of the precipitation change.
- Warming effects on EMF are highly associated with precipitation changes.

## Introduction

Ecosystem functioning and services are being increasingly threated by climate change (Hooper et al., 2005, 2012; Balvanera et al., 2006). Ecosystem multifunctionality (EMF), which simultaneously represents the provision of multiple ecosystem functions, has been proposed as a reliable indicator to reflect the effects of climate change on ecosystem services and functioning (Hector and Bagchi, 2007; Isbell et al., 2011; Manning et al., 2018). Plant and soil microorganisms are major producers and decomposers of terrestrial ecosystems, and have been considered as key drivers in regulating the EMF (Wardle et al., 2004; Bardgett and van der Putten, 2014). Evidence is mounting that global climate change are altering above and belowground biodiversity and which might directly and indirectly influence the EMF (Maestre et al., 2012; Jing et al., 2015; Hu et al., 2021). Understanding how climate change affect the EMF, is important for predicting future ecosystem functions and their provision of services such as productivity, nutrient and carbon cycling.

Plant and soil microbial diversity have been shown to enhance the EMF in grasslands (Maestre et al., 2012; Berdugo et al., 2020; Hu et al., 2021). However, it is noticed that the relationships between biodiversity and EMF depend greatly on the environmental gradient (Isbell et al., 2011; Perkins et al., 2015; Yang et al., 2017). Precipitation is considered as major control on the EMF in grasslands (Maestre et al., 2012; Hu et al., 2021). Higher precipitation generally associated with higher soil water conditions and nutrient availability, which could facilitate resource use by plant and soil microorganisms, and ultimately may attribute to higher EMF (Wu et al., 2012; Jing et al., 2015; Wang et al., 2021a). Several studies have shown that climate warming can directly and indirectly influence plant community, vegetation productivity and soil microbe (Xu et al., 2013; Wang et al., 2021b; Wu et al., 2021), which may attribute to the EMF by altering resources cycling, carbon cycling and other processes involved in the EMF (Zhao et al., 2019; Wang et al., 2021b). However, in grasslands, temperature elevation is often accompanied with precipitation change (Flanagan and Johnson, 2005; Hu et al., 2016). It is likely that warming effects on the EMF may be strongly dependent on precipitation in grassland ecosystems (Jing et al., 2015; Zhao et al., 2019; Hu et al., 2021).

The Tibetan Plateau has become warmer and wetter in recent decades, with the magnitude of climate warming dramatically larger than surrounding areas (Yang et al., 2014; Kuang and Jiao, 2016). The Plateau contains the highest and largest alpine grasslands in the world (Lehnert et al., 2016; Li et al., 2020), which has been regarded sensitive and vulnerable to climate change (Sun et al., 2020). The climate warming with concomitant changes in precipitation is expected to have remarkable effects on the EMF of alpine grasslands by altering ecosystem functions such as vegetation growth, biological diversity and biogeochemical processes (Li et al., 2016b; Ma et al., 2017; Yu et al., 2019; Wang et al., 2020a). Considerable studies have highlighted the responses of EMF to nitrogen addition (Cui et al., 2020; Liu et al., 2021), livestock grazing (Wang et al., 2020b) and environmental changes (Jing et al., 2015; Wang et al., 2021a) in alpine grasslands. Although previous studies have explored the linkages between biodiversity and EMF and further demonstrated the possible consequences of climate change on the EMF in the alpine grasslands (Jing et al., 2015; Pan et al., 2017; Wang et al., 2021b), little attention has been paid to clarify the interactive effects of temperature and precipitation changes on the EMF in the Tibetan alpine meadows.

To examine the effects of climate change on the EMF in alpine meadows, we performed a reciprocal transplantation experiment along an altitudinal gradient of the Nyaiqentanglha Mountains on the central Tibetan Plateau. We simulated warming treatment expected under future climate change and cooling treatment usually occurred over temperature anomalies events through transplanting meadow blocks to lower and higher altitudes (Zhao et al., 2018; Wang et al., 2019a,b). Previous studies have provided basic ecological information along the altitudinal gradient of the Nyaiqentanglha Mountains including plant phenology (Li et al., 2016a, 2020), species distribution (Li et al., 2013; Wang et al., 2013) and carbon dynamics (Zhao et al., 2016, 2018). In this study, we evaluated how the EMF of alpine meadows would response to temperature and precipitation changes across altitudes. Our study aimed to address the following issues: (1) the different response patterns of the EMF to warming and cooling and (2) the underlying mechanisms of precipitation mediate the EMF responses to temperature changes.

## Materials and Methods

### Study Sites

**Figure 1:**
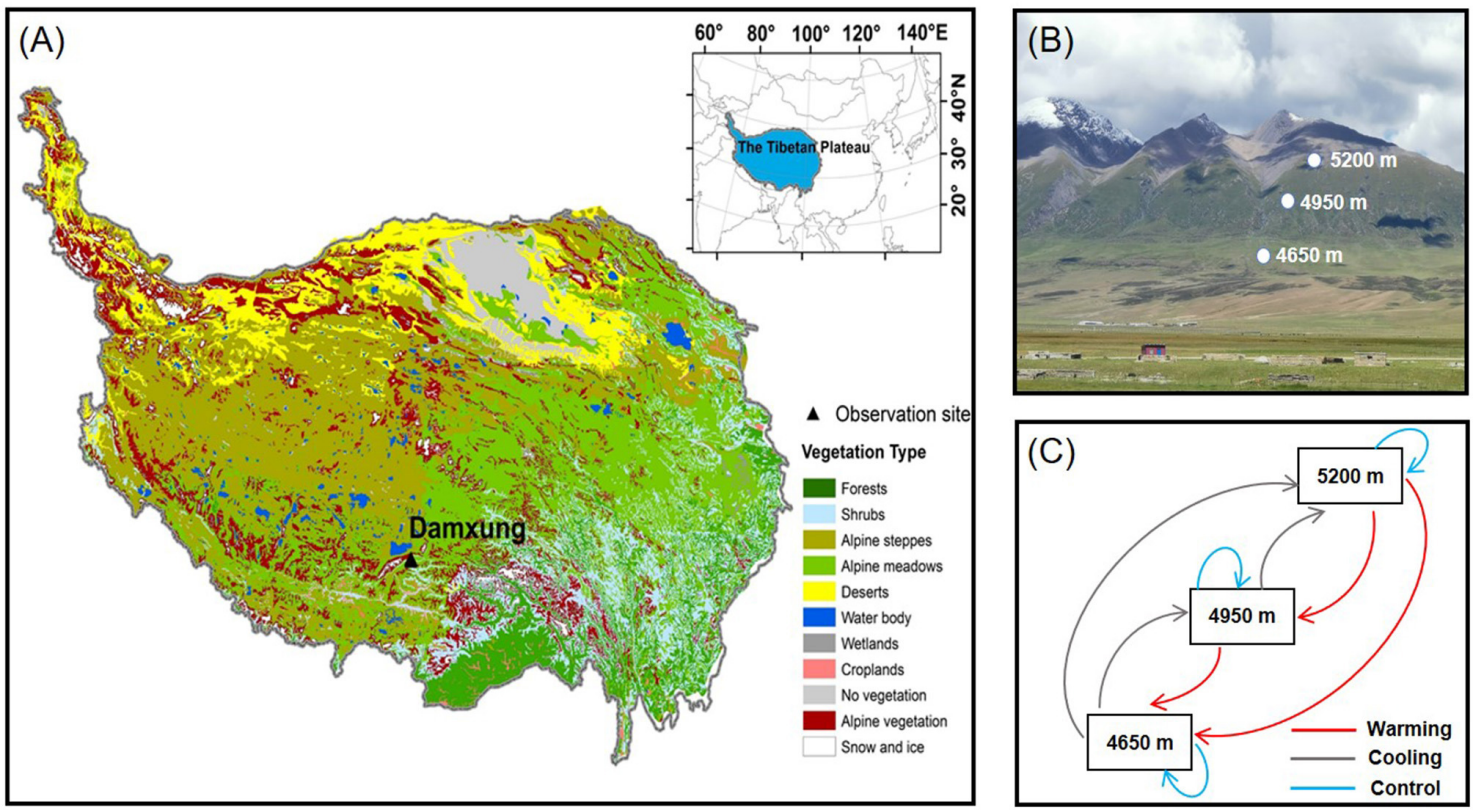
**(A)** Vegetation map of the Tibetan Plateau and the location of study site. **(B)** Diagram of the landscape and experimental sites along the slope of Nyaiqentanglha Mountains. **(C)** Reciprocal transplants experiment along an altitudinal gradient in the Tibetan alpine meadows.

The experiment was conducted at a slope of the Nyaiqentanglha Mountains (30°30′-30°32′N, 91°03′E; 4,650, 4,950, and 5,200 m) on the central Tibetan Plateau (Figure 1; Supplementary Figure S1). This region has a semi-arid climate, characterized by Indian monsoon in summer and the westerlies in winter. Annual precipitation was 479 mm and annual mean air temperature was 1.8°C (Wang et al., 2013). Along the south slope of the Nyaiqentanglha Mountains, annual precipitation increased with increasing altitude, but annual mean air temperature and soil temperature decreased with increasing altitude (Supplementary Figure S2). Alpine meadows dominated by *Kobresia pygmaea* distributed at altitudes between 4,650 m (lower limit) and 5,200 m (upper limit), with a distribution center at 4,950 m. Other coexisting species mainly included *Kobresia humilis, Kobresia humilis, Androsace tapete, Polygonum macrophyllum, Thalictrum alpinum* etc. (Zhao et al., 2016). The vegetation cover at the studies sites is about 50–90% and the alpine meadows generally turn green in May and the plant biomass reach its peak in August. The soils were poorly developed characterized by low clay content within surface soil horizons and the soil types changed from alpine steppe soil to alpine meadow soil along the altitudinal gradient. HOBO weather stations (Onset Inc., Bourne, MA, USA) were set up along the altitudinal gradient from 4,400 to 5,200 m. Air temperature and precipitation (1.5 m aboveground) were recorded by the HOBO data logger to characterize altitudinal environments along the slope.

### Experimental Design

Intact meadow blocks (70 cm × 70 cm wide × 40 cm depth) with attached vegetation were cut off for reciprocal transplantation from the plots at 4,650, 4,950, and 5,200 m in September of 2011 (Figure 1). There were six replicate intact meadow blocks from each altitude, which were randomly transferred throughout the study site. For each of the 3 altitudes, 6 of the 18 intact meadow blocks were reinstated at the same altitude as control (home sites), and the other 12 intact meadow blocks were transferred equally between the other 2 altitudes (translocated plots). Thus, 54 intact meadow blocks (18 soil blocks per altitude × 3 altitudes) were used in the experiment. Compared with the home sites, the meadow blocks transferred from higher altitudes to lower altitudes (translocated sites) can expose to warmer conditions (warming) and transferred from lower altitudes to higher altitudes expose to cooler conditions (cooling). The experimental design was described elsewhere in detail (Zhao et al., 2018).

### Vegetation Measurement

In Mid-August of 2014, we sampled a 50 × 50 cm quadrat within each meadow block and measured the vegetation cover (VC) and community height (CH) at each quadrat. The species richness (SR) was defined according to the number of plant species. Aboveground plant biomass (AGB) was estimated using a non-destructive sampling method, and the detailed information was described elsewhere (Wang et al., 2013; Zhao et al., 2018). The belowground plant biomass (BGB) was estimated through collecting five soil cores (diameter 3.0 cm; depth 10 cm) and washing off the soil by a 2-mm sieve. The BGB samples were dried by an oven at 65°C for 48 h and weighed. The Shannon-Wiener index was calculated to quantify species diversity (SD) of alpine meadow using the following function: $SD=\sum_{i=1}^{S}\left(PiLnPi\right)$, where S is the total number of alpine meadow community and *Pi* is the relative importance value (IV) of the *i*th species calculated as: IV = (relative height + relative cover + relative biomass)/3 (Wu et al., 2009).

### Ecosystem Respiration and Physiochemical Data Measurements

On sunny days during the growing season (June–September), diurnal variation (08:00–18:00, local time) of ecosystem respiration (Re) at 2 h intervals were measured twice a month in 2014 using the opaque chamber of Li-8100 103 automatic soil CO_2_ flux system (LI-COR Biosciences, Lincoln, NE, USA). Soil temperature (ST) and soil moisture (SM) at each collar were measured simultaneously with Re by a Time Domain Reflectometer. Top soil samples (0–10 cm in depth) were collected with a soil auger (diameter: 3.0 cm) within each quadrat. Soil samples for measuring soil organic carbon (SOC) and soil total nitrogen (STN) were air-dried at room temperature and sieved (2–mm mesh). Soil samples for measuring soil microbial biomass carbon (MBC) and soil microbial biomass nitrogen (MBN), ammonium nitrogen (${\text{NH}}_{4}^{+}-\text{N}$) and nitrate nitrogen (${\text{NO}}_{3}^{–}-\text{N}$) analysis were immediately stored in the lab at −20°C until processing. The SOC was measured by the Walkley and Black dichromate oxidation method (Walkley and Black, 1934). The determination of STN was based on the Kjeldahl method (Gallaher et al., 1976). Soil inorganic nitrogen Soil ammonium nitrogen (${\text{NH}}_{4}^{+}-\text{N}$) and nitrate nitrogen (${\text{NO}}_{3}^{–}-\text{N}$) were measured with an auto-analyser (Bran Luebbe, Germany) in 0.05 M K_2_SO_4_ extracts. The MBC and MBN were measured using the chloroform fumigation-extraction method (Vance et al., 1987).

### Ecosystem Multifunctionality

To determine the average multifunctionality, we calculated Z-scores of the 15 ecosystem functions (VC, CH, SR, PD, AGB, BGB, SOC, STN, MBC, MBN, ${\text{NH}}_{4}^{+}-\text{N}$, ${\text{NO}}_{3}^{–}-\text{N}$, ST, SM, Re) evaluated before the analysis, and considered the EMF index as the average Z-score of all the 15 ecosystem functions measured within the plot (Maestre et al., 2012): $EMF=\sum_{i}^{N}\left(Zij/N\right)$, where Z*ij* represents Z-score of the *i*th ecosystem function in the *j*th plot and N is the total number of ecosystem functions evaluated (Wang et al., 2021b).

### Statistical Analysis

One-way analysis of variance (ANOVA) and the Tukey-HSD test was applied to assess the differences in the VC, CH, SR, PD, AGB, ST, SM, SOC, STN, MBC, MBN, ${\text{NH}}_{4}^{+}-\text{N}$, ${\text{NO}}_{3}^{–}-\text{N}$, Re, and the EMF at home sites among the three altitudes (4,650, 4,950, and 5,200 m). Independent sample *t*-test was used to test the differences in these plant and soil functions as well as the EMF between control and transplanted plots. The relative changes (Δ) in the ecosystem functions and the EMF between the control and transplanted plots was calculated as: Relative change (Δ) = Transplanted – Control. Pearson correlation and a simple linear model (y = a + bx) was used to determine correlations of the relative changes in the ecosystem functions with that of the EMF. All statistical analyses were performed using SPSS 20.0 (SPSS Inc., Chicago, Illinois, USA), and graphics were drawn using the OriginPro 2019 software (OriginLab Corporation, Northampton, MA, USA).

## Results

### Vegetation Characteristics

**Figure 2:**
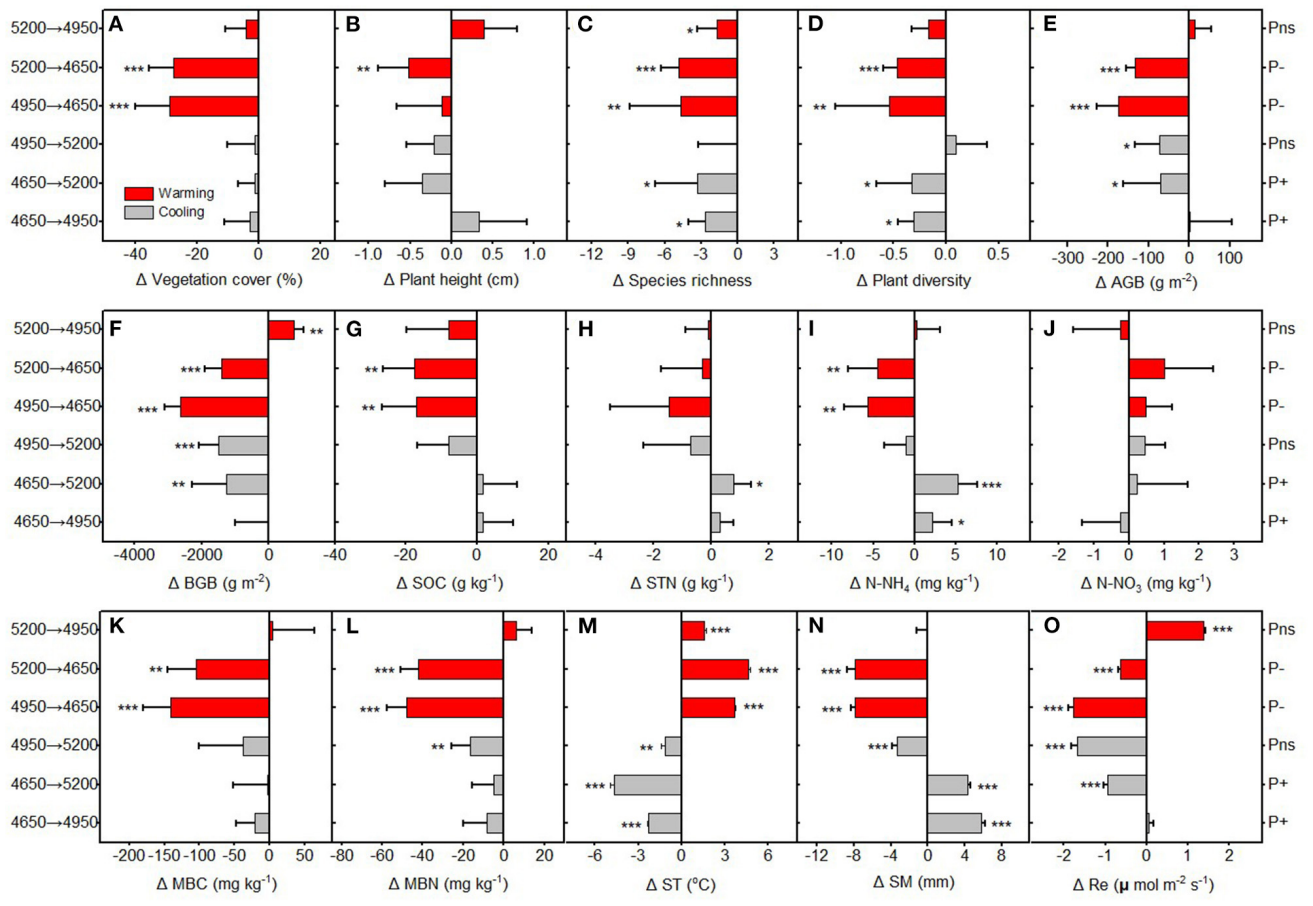
The relative changes in **(A)** vegetation cover (VC), **(B)** community height (CH), **(C)** species richness (SR), **(D)** plant diversity (PD), **(E)** above ground biomass (AGB), **(F)** belowground ground biomass (BGB), **(G)** soil organic carbon (SOC), **(H)** soil total nitrogen (STN), **(I)** ammonium nitrogen (${\text{NH}}_{4}^{+}-\text{N}$), **(J)** nitrate nitrogen (${\text{NO}}_{3}^{–}-\text{N}$), **(K)** soil microbial biomass carbon (MBC) and **(L)** soil microbialbiomass nitrogen (MBN), **(M)** soil temperature (ST), **(N)** soil moisture (SM) and **(O)** ecosystem respiration (Re) between translocated sites and control sites (home-sites) along the altitudinal gradient. P+, precipitation increased; P−, precipitation decreased; Pns, precipitation unchanged. Asterisks are significant at different levels between translocated sites and control sites. *p < 0.05; **p < 0.01; ***p < 0.001. Mean±SD is shown in errorbars.

The CH at the home sites generally decreased with increasing altitude from 4,650 to 5,200 m, and there were no significant differences in the SR and PD at home sites among three altitudes (Supplementary Figure S3). However, we found that VC, AGB, and BGB home site were greatly higher at 4,950 m than that at 4,650 and 5,200 m (Supplementary Figure S3). Both the upward transplantation (cooling) and downward transplantation (warming) resulted in a decrease of the VC, CH, and SR, as compared with the home sites (Figures 2A,C,D). Cooling resulted in a decrease of the AGB and BGB regardless of the precipitation change (Figures 2E,F). However, warming significantly decreased AGB and BGB at the drier destination sites (transplanted from 5,200 and 4,950 m to 4,650 m), but increased BGB at the wetter destination site (transplanted from 5,200 to 4,950 m) (Figures 2E,F).

### Soil Properties and Ecosystem Respiration

The SOC, STN, MBC, and MBN at the home sites were significant higher at 4,950 m compared with that at 4,650 and 5,200 m (Supplementary Figure S3). The ${\text{NO}}_{3}^{–}-\text{N}$ at the home sites generally decreased with increasing altitude (Supplementary Figure S3). However, ${\text{NH}}_{4}^{+}-\text{N}$ were significantly lower at 4,650 m than that at 4,950 and 5,200 m (Supplementary Figure S3). Compared with the home sites, cooling had no significant effects on SOC, MBC, and MBN, whereas experimental warming significantly decreased SOC, MBC, and MBN as downward transplanted to the drier destination sites (transplanted from 5,200 and 4,950 m to 4,650 m) (Figures 2G,K,L). Warming tended to decrease ${\text{NH}}_{4}^{+}-\text{N}$ at drier destination sites, but cooling has a trend to increase ${\text{NH}}_{4}^{+}-\text{N}$ at wetter destination sites (Figure 2I). Both the upward transplantation (cooling) and downward transplantation (warming) have no significant effects on STN and ${\text{NO}}_{3}^{–}-\text{N}$, as compared with the relative home sites (Figures 2H,J). The ST significantly decreased with increasing altitudes (Supplementary Figure S3). However, SM were significant lower at 4,650 m than that at 4,950 and 5,200 m (Supplementary Figure S3). The growing season Re at the home sites was the highest at 4,950 m (Supplementary Figure S3). Cooling generally decreased Re as compared with the relative home sites (Figure 2O). Warming significantly decreased the Re as downward transplanted to the relative drier destination sites, but increased the Re as downward transplanted to the relative wetter destination site (Figure 2O).

### Ecosystem Multifunctionality

**Figure 3:**
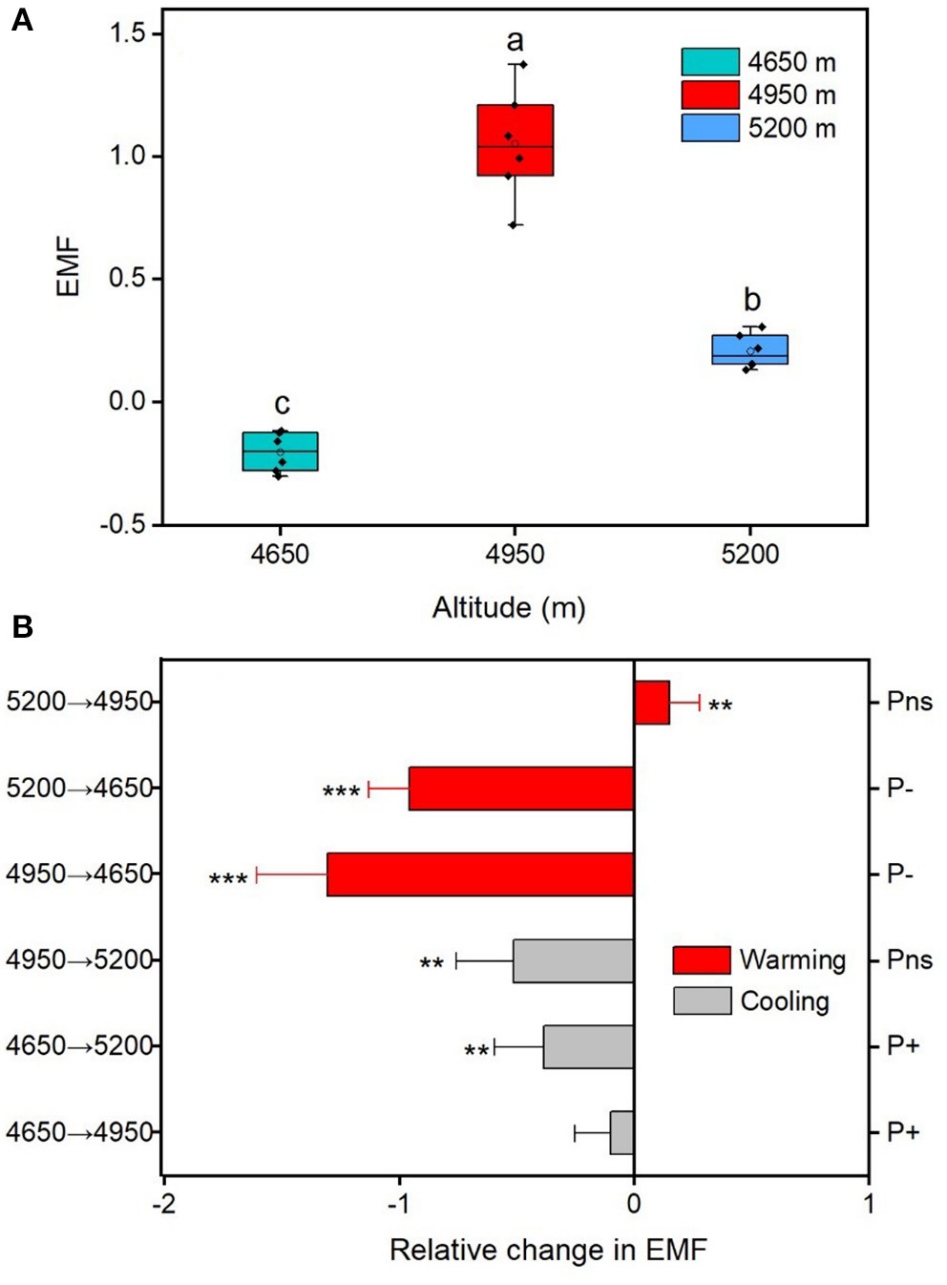
Characteristics of ecosystem multifunctionality (EMF) in alpine meadows. **(A)** Difference of EMF for the home sites across the three altitudes. **(B)** The relative changes in EMF between translocated sites and control sites (home-sites). Asterisks are significant at different levels between translocated sites and control sites. **p < 0.01; ***p < 0.001. Different letters between altitudes indicate the significant difference at 0.05 level. Values in parentheses indicate SD of mean.

**Figure 4:**
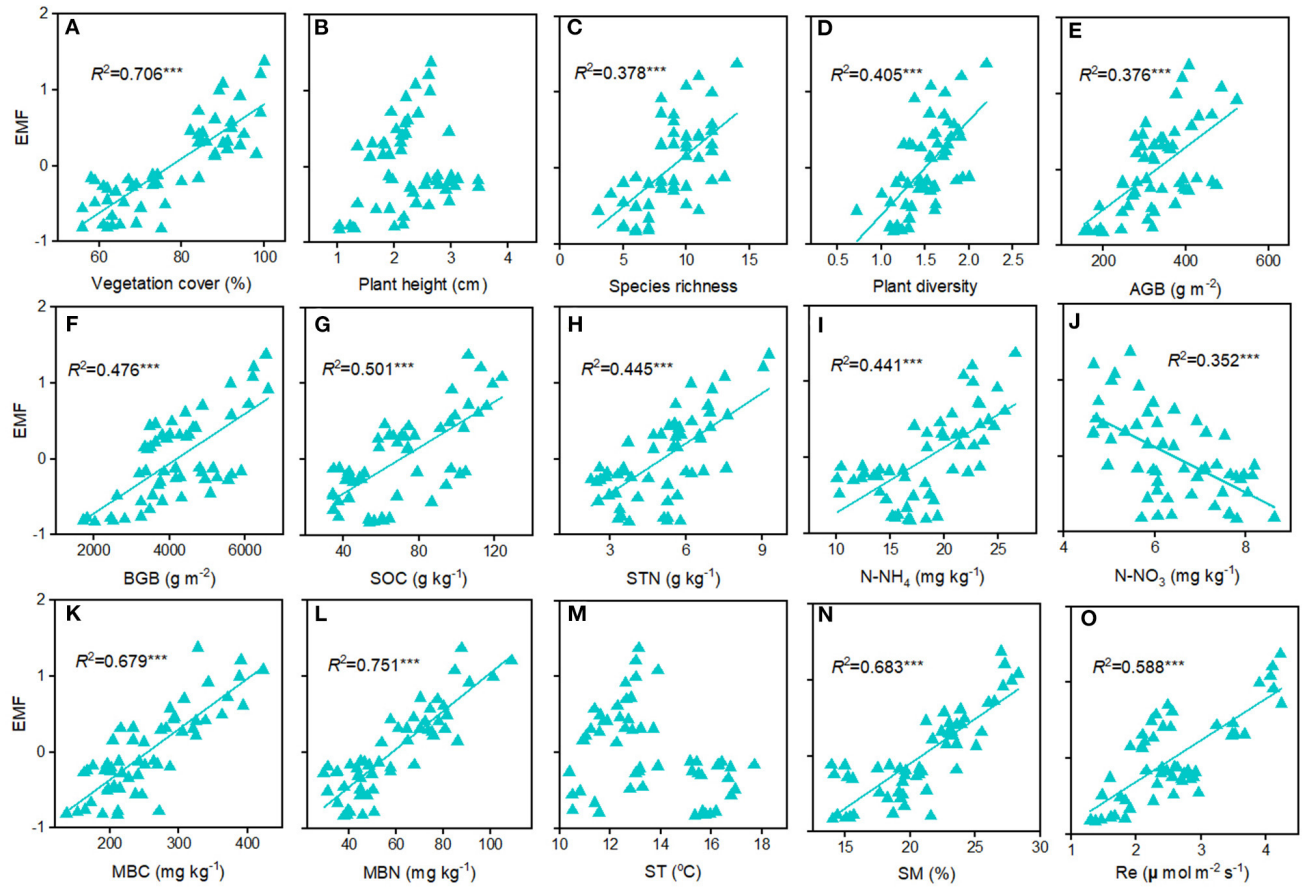
Relationships among ecosystem multifunctionality and factors in the Tibetan alpine meadows. **(A)** VC, vegetation cover; **(B)** CH, community height; **(C)** PD, plant diversity; **(D)** SR, species richness; **(E)** AGB, above ground biomass; **(F)** BGB, belowground ground biomass; **(G)** SOC, soil organic carbon; **(H)** STN, soil total nitrogen; **(I)** ${\text{NH}}_{4}^{+}-\text{N}$, ammonium nitrogen; **(J)** ${\text{NO}}_{3}^{–}-\text{N}$, nitrate nitrogen; **(K)** MBC, soil microbial biomass carbon; **(L)** MBN, soil microbial biomass nitrogen; **(M)** ST, soil temperature; **(N)** SM, soil moisture; **(O)** Re, ecosystem respiration. ***p < 0.001.

**Figure 5:**
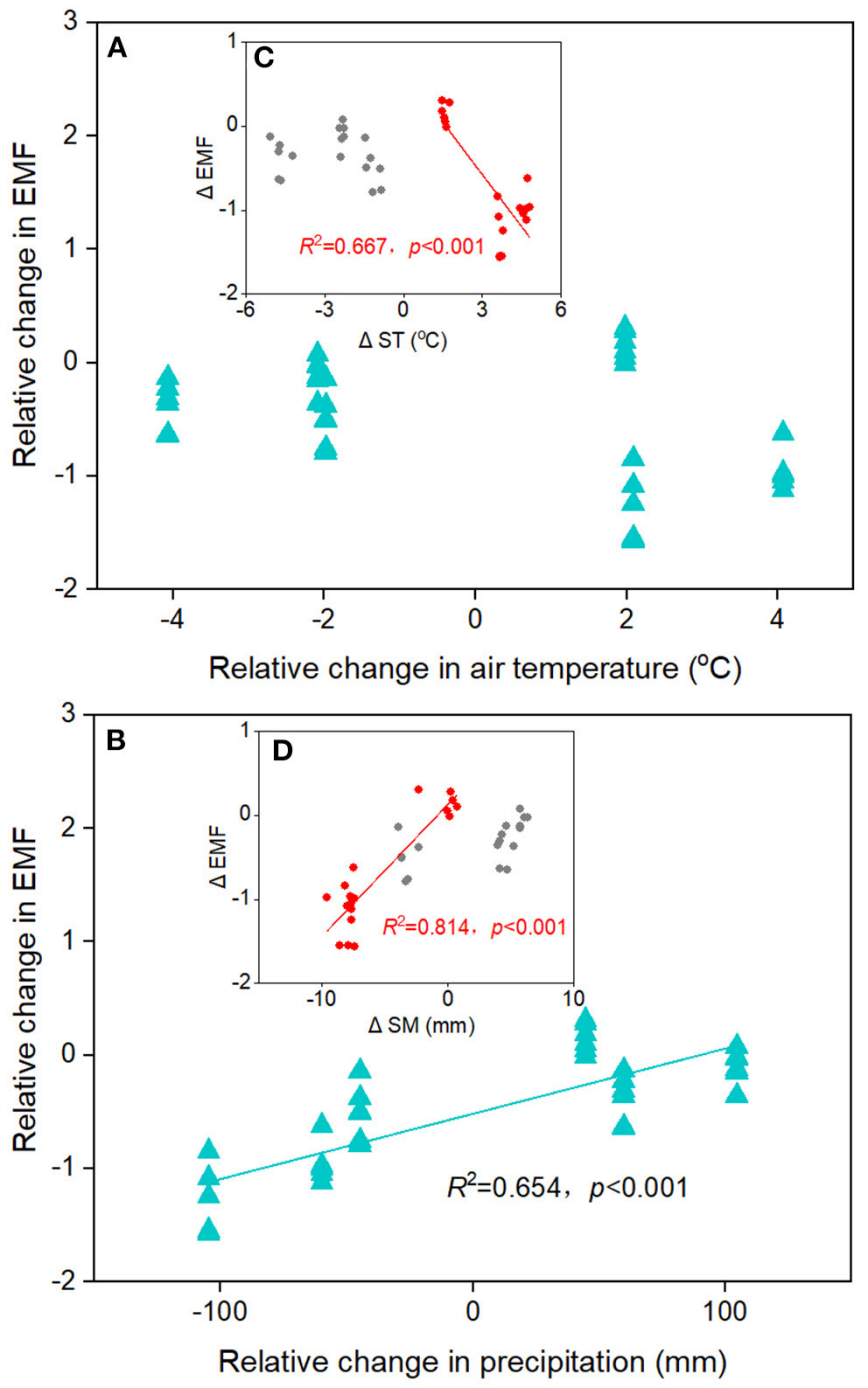
The relationships between transplant-induced changes in **(A)** air temperature and **(B)** precipitation with ecosystem multifunctionality (EMF) in alpine meadows. Insert: the relationships between transplant-induced changes in **(C)** soil temperature (ST) and **(D)** soil moisture (SM) with EMF.

The EMF index at the home sites varies greatly along altitudinal gradient, with the highest value at the 4,950 m and the lowest value at 4,650 m (Figure 3A). The responses of EMF index to warming and cooling also differed remarkable with altitudes. Regardless of the precipitation change, experimental cooling generally resulted in a decrease of the EMF (Figure 3B). However, experimental warming tended to decrease the EMF as downward transplanted to the drier sites but increase the EMF when downward transplanted to the wetter site (Figure 3B). We fitted a linear model to evaluate the relationships between multiple biotic and abiotic factors and the EMF (Figure 4). We found that VC, SR, PD, vegetation productivity (AGB and BGB), SOC, STN, ${\text{NH}}_{4}^{+}-\text{N}$, MBC, MBN, SM, and Re were positively related with the EMF, whereas ${\text{NO}}_{3}^{–}-\text{N}$ was negatively associated with the EMF across altitudes (Figure 4). We found that transplant-induced changes in most of the plant and soil functions were greatly attributed to that of the EMF (Supplementary Figure S4A), but only the transplant-induced changes in CH, AGB, BGB, STN, and SM were significantly associated with that of the EMF (Supplementary Figure S4B). The annual air temperature and ST showed a weakly association with the EMF, but warming-induced changes in ST was significantly and negatively correlated with that of the EMF (Figures 4M, 5A,C). Additionally, the annual precipitation and SM showed a positively association with the EMF, and warming-induced relatively changes in SM was significantly and positively correlated with that of EMF (Supplementary Figure S4; Figures 5B,D).

## Discussions

In alpine grasslands, numerous studies have explored the relationships among plant or soil microbial diversity and the EMF in alpine grasslands and further demonstrated that aboveground or belowground biodiversity generally positively related to the EMF (Jing et al., 2015; Wang et al., 2021a). Our study, along an altitudinal gradient on the central Tibetan Plateau, also found that vegetation productivity and plant diversity were positively correlated with the EMF in alpine meadows (Figures 4C–F). In grasslands, MBC and MBN were strongly linked to soil microbial activity and usually were treated as indicators of nutrient availability for soil microorganisms (Bastida et al., 2016). Likewise, in the present study, the best-fitting relationships were found between the EMF and MBC and MBN (Figures 4K,L), suggesting that belowground biodiversity may be positively correlated with the EMF. However, we also found that transplant-induced changes in the SM rather than ST could well explain that of the EMF (Figure 4N; Supplementary Figure S4B), which indicate that the positive relationships between biodiversity and the EMF in alpine meadows could be particularly modified by precipitation changes.

Climatic changes on the EMF are particularly important in alpine meadows because their biological activity is mainly driven by temperature and precipitation (Zhao et al., 2019), which can affect the EMF through influencing plants and soil microorganisms (Zhao et al., 2019; Wang et al., 2021b). Previous study suggested that the annual mean temperature and total precipitation on the Tibetan Plateau are predicted to increase by 2.8–4.9°C and by 15–21%, respectively, by the end of the twenty-first century (Gao et al., 2014). Ongoing climate warming is likely to reduce aboveground and belowground biodiversity, which will negatively affect the EMF in semi-arid alpine grasslands (Fu et al., 2012; Hu et al., 2020). Our results suggested that experimental warming tended to decrease plant species, vegetation production, MBC and MBN as well as the EMF at the low-precipitation sites but increase those factors at the high-precipitation site (Figure 2). It is possible that plant growth and soil microbial activity might be limited by soil water and nutrient conditions under low precipitation, while under high precipitation, plant and soil microorganisms might play positive roles on the EMF, as observed under the experimental warming (Figure 4; Supplementary Figure S4). In the present study, we found that species richness, plant diversity, vegetation productivity, MBC and MBN were positively correlated with the EMF (Figures 4C,D), the increased EMF under warming can be partly attributed to the increase of soil microbe induced by the higher water conditions and nutrient availability after the transplantation. As reported by the previous studies (Wang et al., 2013; Zhu et al., 2017), the Tibetan alpine vegetation usually suffered from water deficiency due to high evapotranspiration, which might be further aggravated in the semi-arid regions. Reflecting this, the decline of species richness, plant diversity and EMF under downward transplantation (experimental warming) was probably due to the warming-induced drought stress. On the other hand, experimental cooling generally tended to decrease the EMF in alpine meadows (Figure 3B). In high-altitude grasslands, especially for the upper limit of alpine meadows above 5,200 m, low temperature often limits water or nutrients availability and restrains plant growth and soil microbial activity (Wang et al., 2013; Li et al., 2013), that may further attribute to a decrease of the EMF (Figure 3B). This result suggests that temperature rather than precipitation might be the major factor for the decreased EMF of high-altitude (above 4,500 m) alpine meadows under the experimental cooling.

Precipitation and soil moisture are important drivers of the EMF in alpine grasslands (Jing et al., 2015; Pan et al., 2017). Previous studies have showed that changes in precipitation can directly influence plant and soil microbial diversity as well as the EMF, especially in water-limited ecosystems (Liu et al., 2009; Hu et al., 2021). We found that precipitation was significantly associated with the EMF, and warming effects on the EMF were highly dependent on precipitation (Figures 3B, 5B). Such positive relationship between precipitation and the EMF found in the present study is consistent with observed results in alpine grasslands (Jing et al., 2015). The inconsistent effects of experimental warming on the EMF under lower or higher precipitation conditions highlight the importance of precipitation changes on the EMF, especially in the water-limited ecosystems. In grassland ecosystems, higher precipitation and are generally associated with higher soil water conditions and nutrient availability, which could facilitate plant growth and soil microbial activity (Hu et al., 2021; Wang et al., 2021a). The increased precipitation could ameliorate water deficiency and minimize biodiversity losses in semi-arid alpine ecosystems and potentially resulted in increase of the EMF. We also found that precipitation reduction generally resulted in a decrease of the EMF (Figure 3B), which is possibly due to the limitation of microbial activities or the decrease of plant species caused by drought stress. However, the effects of precipitation change on the EMF also largely depended on the temperature environments. In the high-altitude grasslands, where low temperature becomes a limiting factor for plant growth and soil microbial activity, we found that the changes of precipitation would have little influence on the EMF. However, in future, studies are still needed to assess the critical point at which temperature and precipitation both limit the EMF and how the threshold point varies with climate change.

## Conclusion

Our work showed that experimental warming by the downward transplantation tended to increase the EMF when meadow blocks were transplanted to the high-precipitation sites but decrease the EMF as meadow blocks were transplanted to the low-precipitation sites. However, the experimental cooling by the upward transplantation consistently reduced the EMF regardless of the precipitation change. Further analysis showed that the increase of the EMF under experimental warming was closely related to the increase of plant species and biomass, whereas the reduction of EMF under cooling was highly correlated with the decrease of soil temperature. The increase of EMF under the experimental warming was closely related to the variation of both plant and soil functions, whereas the reduction of EMF under the cooling was highly correlated with the decrease of plant function. Our study provides insight into how the EMF of alpine meadows will response to warming expected under future climate change and to cooling that usually occurred over temperature anomalies events.

## Data Availability Statement

The original contributions presented in the study are included in the article/Supplementary Material, further inquiries can be directed to the corresponding author.

## Author Contributions

YM, GQ, and JZ: investigation and writing. GQ and LT: conceptualization and methodology. WW and JZ: analyzed the data. LT and JZ: review and editing. All authors contributed to the article and approved the submitted version.

## Funding

This work was funded by the Second Tibetan Plateau Scientific Expedition and Research Program (2019QZKK0106), the National Natural Science Foundation of China (42071058, 91837312, and 41701276), and a grant from Grassland Science Institute, Tibet Academy of Agricultural and Animal Husbandry Sciences (CYS-TC-2021-001).

## Conflict of Interest

The authors declare that the research was conducted in the absence of any commercial or financial relationships that could be construed as a potential conflict of interest.

## Publisher's Note

All claims expressed in this article are solely those of the authors and do not necessarily represent those of their affiliated organizations, or those of the publisher, the editors and the reviewers. Any product that may be evaluated in this article, or claim that may be made by its manufacturer, is not guaranteed or endorsed by the publisher.
