# Supplementary material for: Precipitation Alters the Effects of Temperature on the Ecosystem Multifunctionality in Alpine Meadows
Source: Front Plant Sci. 2022 Feb 9;12:824296. doi: 10.3389/fpls.2021.824296 (PMC8864076; doi:10.3389/fpls.2021.824296)
Supplement: Supplementary file 1 [file Data_Sheet_1.docx]

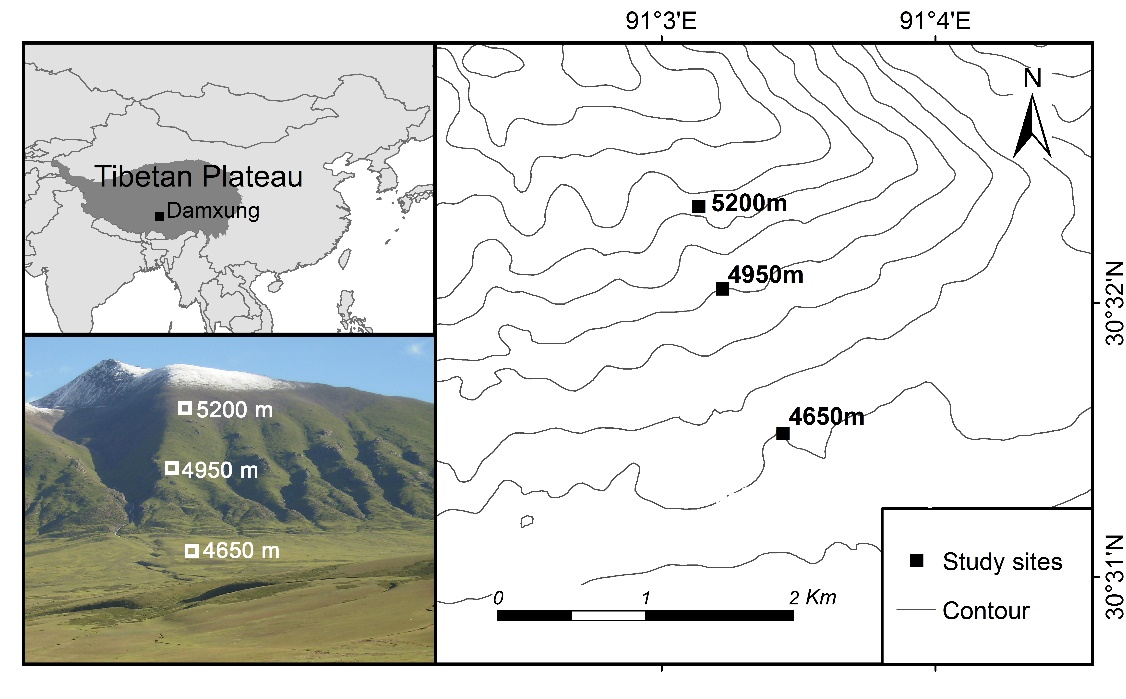


**FIGURE S1** Vegetation map of the Tibetan Plateau and the location of study site.


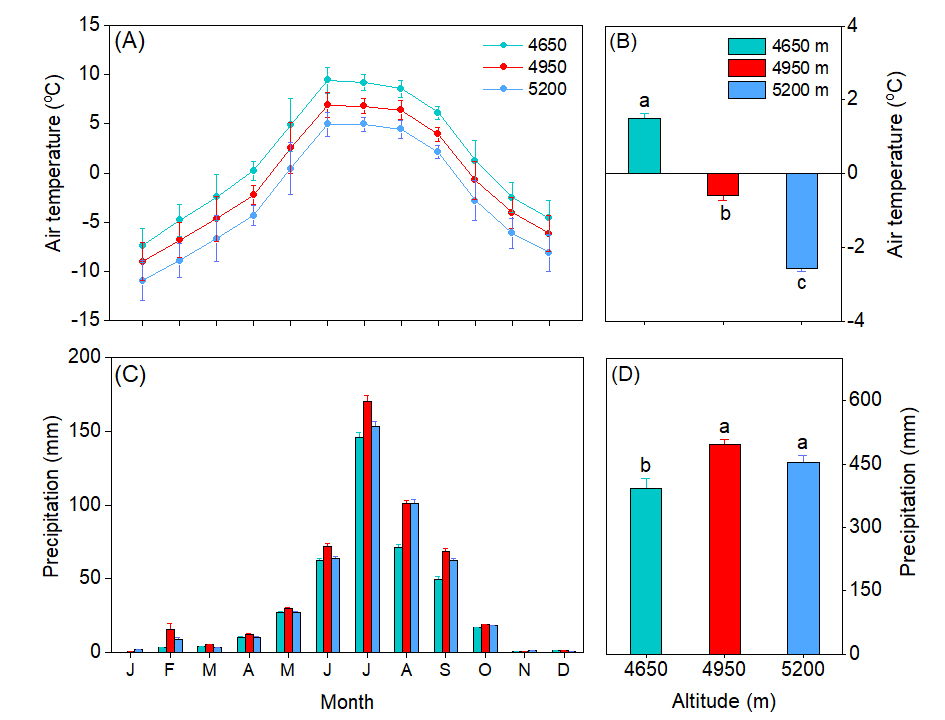


**FIGURE S2** (A) Changes in monthly mean air temperature and (B) average annual air temperature at 4650 m, 4950 m and 5200 m. (C) Dynamics of monthly precipitation and (D) average annual precipitation at 4650 m, 4950 m and 5200 m.


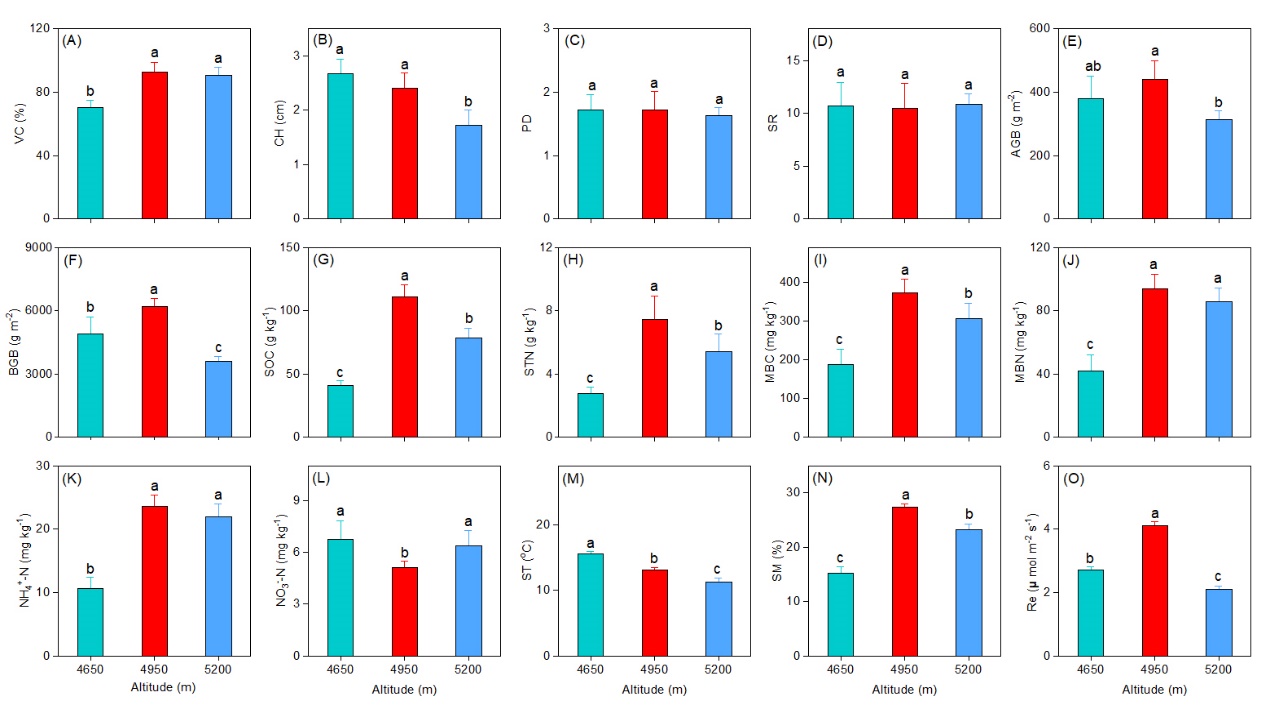


**FIGURE S3** Differences of plant and soil functions for the home sites across the three altitudes. (A) vegetation cover (VC), (B) community height (CH), (C) plant diversity (PD), (D) species richness (SR), (E) aboveground biomass (AGB), (F) belowground ground biomass (BGB), (G)soil organic carbon (SOC), (H) soil total nitrogen (STN), (I) ammonium nitrogen (NH_4_^+^–N), (J) nitrate nitrogen (NO_3_^–^–N), (K) soil microbial biomass carbon (MBC), (L) soil microbial biomass nitrogen (MBN), (M) soil temperature (ST), (N) soil moisture (SM) and (O) ecosystem respiration (Re). Different letters between altitudes indicate the significant difference at 0.05 level. Values in parentheses indicate SD of mean.


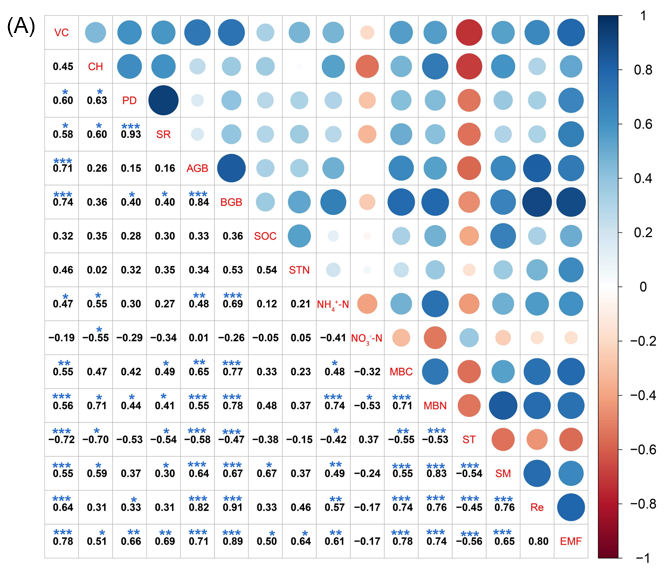
**
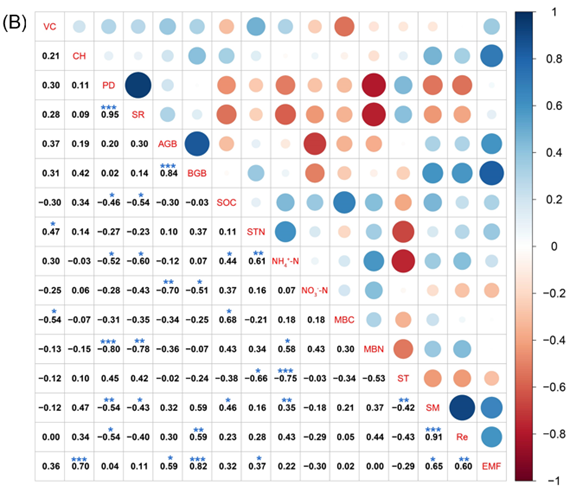
**

**FIGURE S4** Relationships among ecosystem multifunctionality and factors in the Tibetan alpine meadows. VC, vegetation cover; CH, community height; PD, plant diversity; SR, species richness; AGB, aboveground biomass; BGB, belowground ground biomass; SOC, soil organic carbon; STN, soil total nitrogen; NH_4_^+^–N, ammonium nitrogen; NO_3_^–^–N, nitrate nitrogen; MBC, soil microbial biomass carbon; MBN, soil microbial biomass nitrogen; ST, soil temperature; SM, soil moisture; Re, ecosystem respiration. **p* < 0.05; ***p* < 0.01; ****p* < 0.001.
